# Cerebrovascular dysregulation and postoperative cognitive alterations after carotid endarterectomy

**DOI:** 10.1007/s11357-024-01237-6

**Published:** 2024-06-15

**Authors:** Ágnes Dóra Sándor, Zsófia Czinege, András Szabó, Eszter Losoncz, Krisztina Tóth, Zsuzsanna Mihály, Péter Sótonyi, Béla Merkely, Andrea Székely

**Affiliations:** 1 Department of Anesthesiology and Intensive Therapy, Semmelweis University, Budapest, Hungary; 2 Department of Vascular and Endovascular Surgery, Semmelweis University, Budapest, Hungary; 3 Doctoral School of Theoretical and Translational Medicine, Semmelweis University, Budapest, Hungary; 4 Heart and Vascular Center, Semmelweis University, Budapest, Hungary

**Keywords:** Cognitive function, Postoperative neurocognitive disorder, Carotid endarterectomy, Cerebral tissue saturation, Near-infrared spectroscopy

## Abstract

There are controversial data about the effect of carotid endarterectomy regarding postoperative cognitive function. Our aim was to analyze the effect of cerebral tissue saturation monitored by near-infrared spectroscopy (NIRS) on cognitive function. Perioperative data of 103 asymptomatic patients undergoing elective carotid surgery under general anesthesia were analyzed. Preoperatively and 3 months after the operation, MMSE (Mini Mental State Examination) and MoCA (Montreal Cognitive Assessment) tests were conducted. For cerebral monitoring, NIRS was used, and the lowest rSO_2_ value and the degree of desaturation were calculated. Cognitive changes were defined as one standard deviation change from the preoperative test scores, defined as postoperative neurocognitive decline (PNCD) and cognitive improvement (POCI). PNCD was found in 37 patients (35.92%), and POCI was found in 18 patients (17.47%). Female gender, patients with diabetes, and the degree of desaturation were independently associated with PNCD. The degree of desaturation during the cross-clamp period negatively correlated with the change in the MoCA scores (*R* =  − 0.707, *p* = 0.001). The 15.5% desaturation ratio had 86.5% sensitivity and 78.8% specificity for discrimination. For POCI, a desaturation of less than 12.65% had 72.2% sensitivity and 67.1% specificity. POCI was associated with lower preoperative MOCA scores and a lower degree of desaturation. We found a significant relation between the change of postoperative cognitive function proven by the MoCA test and cerebral tissue saturation during the clamping period in patients undergoing carotid endarterectomy.

**Supplementary Information:**

The online version contains supplementary material available at 10.1007/s11357-024-01237-6.

## Introduction

The role of carotid endarterectomy in the prevention of stroke is undebatable, but its effect on cognitive function is controversial [1, 2].

Postoperative cognitive decline is associated with longer hospital stays, impaired mobility, increased need for rehabilitative services, and increased risk of long-term mortality [3, 4]. The development of PNCD (perioperative neurocognitive disorder) after carotid endarterectomy was associated with a fivefold increase in mortality risk [5]. Furthermore, impaired cognitive function makes patients vulnerable and defenseless, and it places a financial burden not only on the patients and their families but also on the whole economy [6].

Age, hypertension, diabetes mellitus, hyperlipidemia, stroke, smoking, and low education level are the most common risk factors for cognitive impairment [7]. As most of these are not preventable, the emphasis must be on those that can be prevented or at least influenced, such as carotid artery stenosis.

Theoretically, removal of a plaque, which may not only be a source of emboli but also the reason for hypoperfusion, should lead to the improvement or at least the preservation of cognitive skills, but in clinical practice, its positive impact is not perceptible in every case [5].

Measurement methods for a more exact quantitative estimation of cognitive function have been sought for decades. The MMSE (Mini-Mental State Examination) and MOCA (Montreal Cognitive Assessment) have been widely used for this purpose. The MMSE has been used for the determination of dementia and been validated and extensively used to separate patients with cognitive impairment from those without it. The MoCA has proven to be an effective screening tool for detecting mild changes in cognitive performance. Both are reliable tools to assess a patient’s cognitive status in both clinical practice and research.

NIRS (near-infrared spectroscopy) is a validated technique to monitor cerebral perfusion [8–10].

The link between cerebral oxygen saturation and cognitive function is still controversial, as the exact limit of potentially harmful desaturation is still not obvious [11–14].

The aim of our study was to investigate the relationship between cerebral oxygen desaturation and changes in cognitive function by evaluating the results of the MOCA and MMSE tests of patients’ undergoing carotid artery surgery. The secondary aim of the study was to compare the absolute value of cerebral saturation measured by NIRS to the relative changes compared to the baseline pre-cross-clamp value and expressed as ratio of desaturation. Furthermore, we aimed to search for factors that can impact cognitive changes.

## Methods

### Participants

We evaluated the changes in regional cerebral tissue saturation of 126 patients who underwent elective carotid endarterectomy at Városmajor Heart and Vascular Center, Semmelweis University, Budapest, between 2019 and 2021 (May 13, 2019–Nov 04, 2021). After providing full information, all the patients gave written consent to participate in the study according to the guidelines approved by the Ethical Committee of Semmelweis University (Semmelweis University Regional and Institutional Committee of Science and Research Ethics, 17/2019, 02/15/2019). The study was performed according to the Declaration of Helsinki, and it was registered on ClinicalTrials.gov (NCT03907943) on Mar 26, 2019.

The indication for endarterectomy was carotid artery stenosis, with stenosis exceeding 70% based on the NASCET (The North American Symptomatic Carotid Endarterectomy Trial) criteria [15]. All of our patients were asymptomatic.

The exclusion criteria were lack of consent, history of dementia, symptomatic carotid artery stenosis, and atrial fibrillation. An MMSE score below 24 points was a contraindication for enrollment, and those who were not able to reach this value were not tested further. The Cronbach’s alpha of our population regarding their MMSE scores was 0.79.

**Fig. 1:**
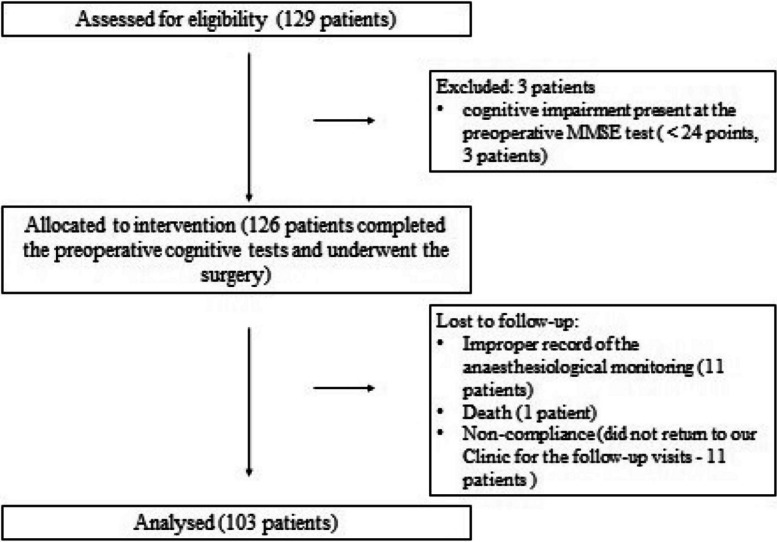
Consort flow diagram of the enrollment and follow-up

The flow diagram of the enrollment and follow-up is shown in Fig. 1. From May 2019 to November 2021, we enrolled 129 patients. All patients completed the MMSE and the MoCA preoperatively. Three patients were excluded due to the presence of cognitive impairment in the preoperative MMSE. In 115 cases, we have the proper record of the anesthesiological monitoring.

Three-month postoperative cognitive assessments were completed in 103 patients. One patient died before the first control (SARS-COVID pneumonia), and 11 patients did not return to our follow-up visit.

The decision about the type of surgery and the indication for shunt use was made by the surgeons based on the patients’ anatomy of the circle of Willis, the condition of the contralateral internal carotid artery (ICA), and the position of the stenosis. The anatomy of the circle of Willis was analyzed and classified as complete (no component was absent or hypoplastic) or incomplete.

All patients underwent balanced general anesthesia. Anesthesia was induced with propofol (2–5 mg/kg or until loss of consciousness) and fentanyl (2–10 mcg/kg). For maintenance, we used sevoflurane (114 patients, 1,8–2 MAC) or isoflurane (12 patients, 1,2–1,4 MAC) and fentanyl (0,02 mg/kg—or as necessary to avoid signs of pain) as analgesic agents. For muscle relaxation, atracurium or rocuronium was used. The choice of the volatile agent and the muscle relaxant was at the discretion of the anesthesiologist. After intubation, all patients were ventilated with 40–50% oxygen, aiming to maintain end-tidal carbon dioxide between 33 and 40 mmHg.

The anesthesia station was a GE Aisys CS^2^. Routine monitoring included electrocardiogram, intra-arterial blood pressure measurement, pulse oximetry, capnography, and entropy. For sufficient depth of anesthesia, state and response entropy values were maintained between 40 and 60 [16]. Before clamping, 2500 IU of heparin was administered to all patients. At the end of the procedure, heparin was reversed with protamine.

Bilateral frontoparietal regional cerebral tissue oxygenation (rSo_2_) was measured using a Somanetics Invos™ 5100C cerebral oximeter. NIRS monitoring was started after arrival to the operating room, continued throughout the whole procedure, and finished just before the patients left the operating room. The sampling frequency was 0.166 Hz. The phases of the operation were accurately marked in all the monitors.

As a baseline value, we determined the median of regional cerebral tissue saturation values of the 2-min-long preclamping (CCA clamping) period.

To prevent the evaluation of measurement errors, which would be misleading, we analyzed not one single value, but we looked for the serial of the lowest values lasting 30 second during the cross-clamp period. For further calculation, we used their median. So, the lowest cross-clamp saturation value was calculated from the median of the lowest saturation values lasting at least 30 second during the cross-clamp period (from clamping the CCA and ICA to the complete removal of the clamp). From these data, we calculated the ratio from baseline to the lowest cross-clamp values as a percentage using the following formula: (rSO_2preclamp_ − rSO_2clamp_)/rSO_2preclamp_ × 100.

The target mean arterial pressure ranged ± 20% of the preoperative level. In cases of hypotension, we used intravenous norepinephrine (0.01–0.1 mcg/kg/min) to maintain the mean arterial pressure at an adequate level, in the targeted range. Those patients who showed bradycardia tendency during vessel preparation received lidocaine infiltration of the surgical area.

### Neuropsychological tests

Patients’ cognitive evaluation for general cognitive impairment detection included the Mini-Mental State Examination (MMSE) and Montreal Cognitive Assessment (MoCA) supervised by the same physician (SA- HU710556625-01).

Neurocognitive testing was performed preoperatively, a day before surgery, and the next survey was scheduled at 3 months after the surgery.

Our survey contained elements to assess patients’ condition with regard to frailty. We had questions aiming to obtain a picture of their medical history, general condition, social situation, level of education and profession, capability of self-care, and, superficially, their psychological condition and history.

Cognitive evaluation started with the MMSE to exclude those suffering from dementia. The cutoff value for the MMSE was 24 points, and those who were not able to reach this value were not tested anymore [17]. The survey continued with a discussion about the frailty parameters and was closed completing the MoCA test. The 8.1 version of the MoCA test was used. Changing the version of the MoCA test made it possible to eliminate the risk of the learning effect.

A decrease in the test scores with a minimum of the standard deviation of the results of the preoperative test or more (MMSE: 1.79, MoCA: 2.28) was considered a cognitive decline (PNCD) and an increase with the same amount as cognitive improvement (POCI) [18].

### Statistical analysis

All statistical analyses were performed using SPSS software (IBM SPSS Statistics Version 20). A *P* value < 0.05 was considered statistically significant. To analyze the distribution of the data, we used the Kolmogorov‒Smirnov test. Normally distributed data are presented as the mean and standard deviation, and non-normally distributed data are presented as the median and interquartile range. Categorical variables are presented as numbers and percentages. For comparing groups, we used the Mann‒Whitney *U* test in the case of non-normally distributed data and Student’s *t* test for normally distributed data. Discrete data were compared using Pearson’s chi-squared test.

To compare the differences among patients with different directions of cognitive change (three groups), we used univariate analysis of variance in cases of normally distributed data, the Kruskal‒Wallis test in cases of non-normally distributed data, and Pearson’s chi-squared test for categorical data. As a post hoc test, we applied the Mann‒Whitney *U* test.

We performed univariate logistic regression analysis for all variables to predict PNCD and POCI. With the multivariable logistic model, we analyzed factors with a *p* value less than or equal to 0.15.

The correlation between the rSO_2_ values, the degree of desaturation during the clamping period, and the change in the scores of the cognitive test were evaluated with Spearman’s correlation analysis.

The best cutoff of rSO_2_ decrease for the detection of intraoperative hypoperfusion causing changes in cognitive performance was analyzed by receiver operating characteristic (ROC) curves. SPSS software (IBM SPSS Statistics Version 20) was used to create figures.

## Results

A total of 103 patients were analyzed, and the baseline characteristics are shown in Table 1. Seventy-four (71.84%) had endarterectomy, and 29 (28.15%) had thromboendarterectomy with the application common to internal carotid artery shunt.

**Table 1:** Baseline characteristics and intraoperative parameters (*N* = 103 patients)

Characteristics	Mean, median, or number	Standard deviation, interquartile range and percentage
Age (mean, SD)	70.49	7.17
Male (N, %)	59	57.28
Vascular POSSUM (median, IQR)	18	16–21.25
BMI (median, IQR)	28.34	25.39–30.44
Smoker (current) (N, %)	31	30.09
Alcohol (N, %)	12	11.65
Hypertension (N, %)	95	92.23
Ischemic heart disease (N, %)	34	33
Previous stroke (N, %)	23	22.33
Diabetes mellitus type 1 (N, %)	11	10.67
Diabetes mellitus type 2 (N, %)	28	27.18
Chronic obstructive lung disease (N, %)	20	19.41
Hyperlipidemia (N, %)	53	51.45
Thyroid dysfunction (N, %)	16	15.53
Peripheral arterial disease (N, %)	22	21.35
Type of the surgery (EEA) (N, %)	74	71.84
Operated side (left) (N, %)	50	48.54
Shunt use (N, %)	29	28.15
Time of the shunted period (median, IQR)	33	25–39.5
Time of the clamping period (median, IQR)	27	23–33.25
Median of the preclamp. rSO_2_ values (mean, SD)	70.64	9.62
Lowest rSO_2_ values during clamping period (mean, SD)	58.9	10.81
Median of the degree of desaturation (median, IQR)	14.45	9.85–23.37
Mean of MAP during the clamping period (mean, SD)	87.54	9.91
Vasopressor use during the clamping period (N, %)	73	70.87

*BMI* body mass index, *EEA* eversion endarterectomy, *IQR* interquartile range, *MAP* mean arterial pressure, *rSO*_*2*_ regional oxygen saturation, *SD* standard deviation, *Vascular POSSUM* Vascular-Physiological and Operative Severity Score for the enUmeration of Mortality and Morbidity

Complications occurred in two patients, of whom one developed postoperative bleeding requiring surgery and one developed a neurological complication (transient ischemic attack, cerebral ischemia, and other intracranial disorders were ruled out) during postoperative hospitalization. None of the patients had cerebral hyperperfusion syndrome.

The median of the desaturation during the cross-clamp period (rSO_2desat_) was 14.45% (9.85–23.37%), and the median of the lowest cerebral tissue saturation (rSO_2low_) value was 59% (51–66%).

Female patients experienced significantly greater desaturation (rSO_2desat_:17.67% IQR: 10.46–28.05%) and had significantly lower cerebral tissue saturation values (rSO_2low_: 55.5% IQR: 48.5–60.75%) than male patients (rSO_2desat_: 13.63% IQR: 9.52–17.64%, *p*: 0.05, rSO_2low_: 62% IQR: 55.5–69%. *p*: 0.002). We did not find any difference regarding the anatomy of the circle of Willis and the degree of stenosis between the sexes.

Significantly greater desaturation was observed in patients with incomplete circle of Willis (rSO_2desat_:16.92% IQR: 11.49–26.13%), compared to patients with complete circle of Willis (rSO_2desat_: 12.53%, IQR: 7.82–16.86%, *p*: 0.029); otherwise, there was no difference between the patients regarding the lowest cerebral tissue saturation values or the degree of desaturation compared to the preclamping period.

### MOCA

**Fig. 2:**
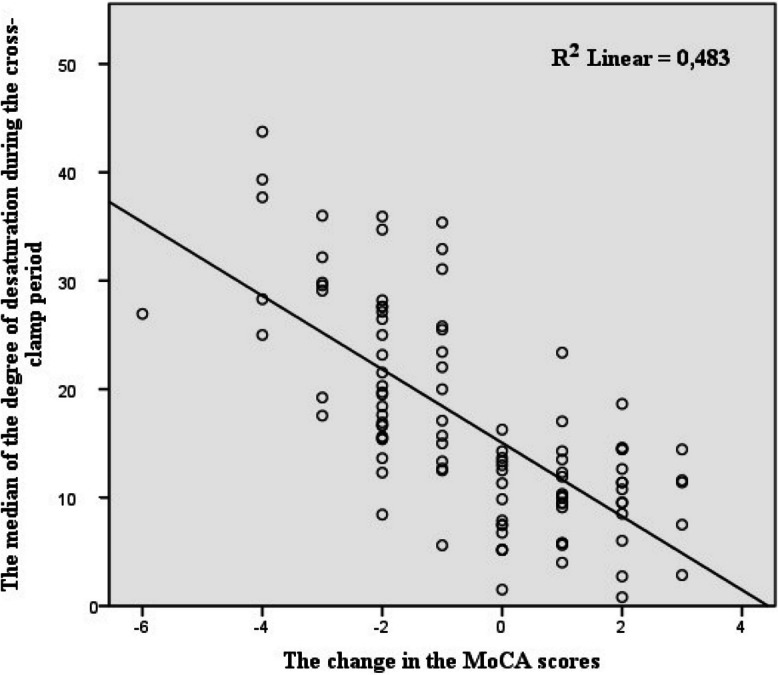
The correlation of the change in the MoCA scores and the degree of desaturation during the cross-clamp period

In terms of the MoCA, the preoperative value was 27 (IQR: 26–29), and the postoperative score was 27 (IQR: 25–28). One SD decline in the MoCA scores was seen in 37 patients, and improvement was observed in 18 patients. There was a statistically significant negative correlation between the value of cerebral desaturation compared to the preclamping values and the change in the MoCA scores at 3 months (R = -0.707, p = 0.001). The results are illustrated in Fig. 2.

We found weaker correlation between the lowest cerebral saturation values and the changes in the MoCA score (*R* = 0.529; *p* = 0.001).

**Fig. 3:**
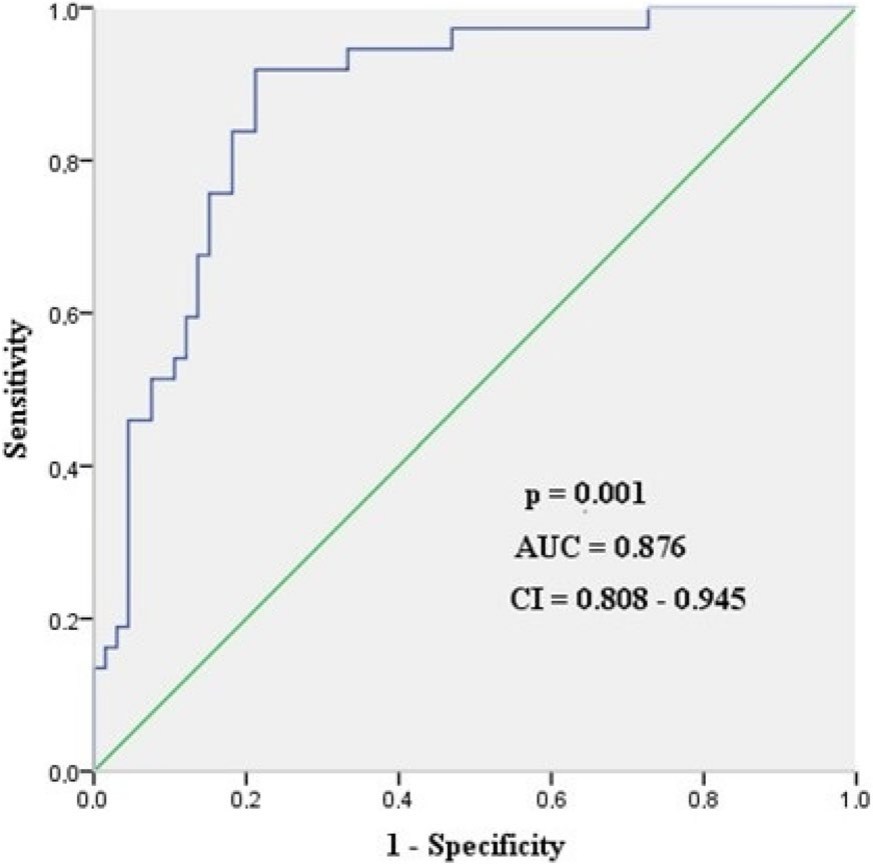
ROC curve of rSO_2_ desaturation for detecting postoperative cognitive decline proven by MoCA test. AUC, area under the curve; CI, confidence interval

Applying ROC analysis, we identified a cutoff of a 15.5% decrease in regional cerebral tissue saturation as a threshold for cognitive decline with a sensitivity of 86.5%, specificity of 78.8%, positive predictive value of 69.6%, negative predictive value of 91.2%, AUC = 0.876, and *p* = 0.001. The results are shown in Fig. 3.

To confirm this connection, we calculated the cumulative duration of time spent below this level of desaturation (≥ 15.5%). It differed significantly between the groups (Kruskal‒Wallis test, *p* = 0.001).

Regarding the lowest cerebral rSO_2_ values, the discriminative value was 57.5% (AUC: 0.794, *p*: 0.001), which had a sensitivity of 75.7%, a specificity of 69.7%, a positive predictive value of 58.3%, and a negative predictive value of 83.6%.

**Fig. 4:**
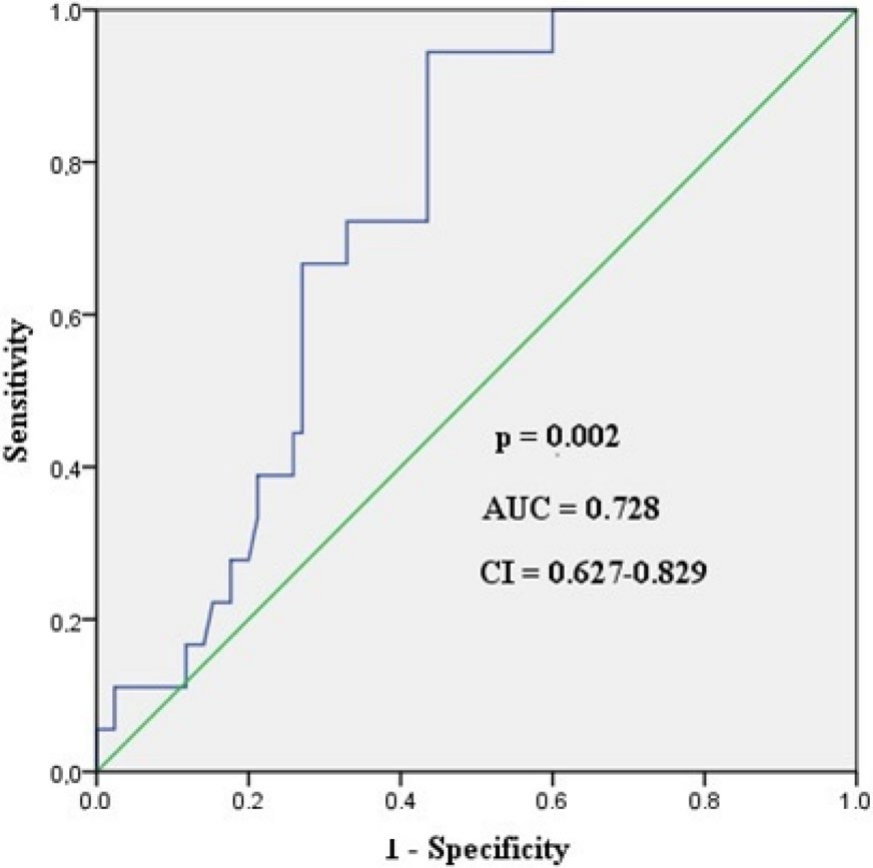
ROC curve of rSO_2_ desaturation for detecting postoperative cognitive improvement proven by MoCA test. AUC, area under the curve; CI, confidence interval

In the case of cognitive improvement, the ROC analysis yielded an AUC of 0.728 (*p*: 0.002). Desaturation equal to or less than 12.65% had a sensitivity of 72.2%, a specificity of 67.1%, a positive predictive value of 31.7%, and a negative predictive value of 91.9%. The ROC curve is shown in Fig. 4.

Regarding the lowest rSO_2_ values, there was a significant but weaker connection with the potential improvement in cognitive function using the MoCA (AUC: 0.653, *p*: 0.042). Picking rSO_2_: 60.75% as a limit, it had a sensitivity of 66.7%, a specificity of 61.2%, a positive predictive value of 26.7%, and a negative predictive value of 89.7%.

### MMSE

The median preoperative MMSE score was 29 (IQR: 27–29), while the postoperative value was 29 (IQR: 28–30). The standard deviation of the MMSE was 1.79, one SD decline at the third month survey was observed in nine patients (8.73%), and improvement (+ 1 SD) was observed in 19 (18.44%) patients. The MMSE score did not show a significant correlation neither with the ratio of desaturation (*R* =  − 0.193, *p* = 0.051) nor with the lowest saturation value (*R* =  − 0.070, *p* = 0.483). The ROC curve and the results are displayed in the Supplement (Fig s1., Fig s2.).

### Factors associated with MOCA score decline and improvement

Building upon our further results, we created three groups: patients with postoperative cognitive decline (PNCD group) confirmed by the MoCA, patients with postoperative cognitive improvement (POCI group) confirmed by the same test and patients with no change in cognitive status proven by the MoCA (NCCF group). PNCD was found in 37 patients (35.92%), and POCI was found in 18 patients (17.47%). The comparison of the three groups is summarized in Table 2.

**Table 2:** Comparison of demographic and clinical factors between the three groups created based on the changes in the MoCA scores

Characteristic	NCCF group (48 patients, 46.6%)		PNCD group (37 patients, 35.92%)		POCI group (18 patients, 17.47%)		*p* value
Age (y, mean, SD)	69.29	7.64	71.31	6.76	72	6.49	0.274
Male (N, %)	33	68.80	14	37.83	12	66.66	0.011
Vascular POSSUM (median, IQR)	18	15.25–21	19	17–23	18	16–22.25	0.344
BMI (median, IQR)	27.61	24–30.46	28.39	26.67–30.54	28.5	26.07–29.75	0.622
Smoker (N, %)	18	37.5	11	29.72	2	11.11	0.114
Alcohol (N, %)	7	14.58	2	5.4	3	16.66	0.326
Hypertension (N, %)	44	91.66	34	91.89	17	94.44	0.928
Systolic blood pressure (mmHg, mean, SD)	145.95	21.47	147.13	24.52	148.53	19.41	0.924
Diastolic blood pressure (mmHg, mean, SD)	78.52	12.87	80.63	13.87	84	9.16	0.205
Mean arterial pressure (mmHg, mean, SD)	101	13.48	102.80	15.87	105.51	11.54	0.563
Ischemic heart disease (N, %)	17	35.41	12	32.43	5	27.77	0.838
Previous stroke (N, %)	11	22.91	8	21.62	4	22.22	0.99
Diabetes mellitus type 1 (N, %)	3	6.25	4	10.81	2	11.11	0.336
Diabetes mellitus type 2 (N, %)	13	27.08	13	35.13	1	5.55	0.041
Diabetes mellitus type 1 + type 2 (N, %)	16	33.33	17	45.94	3	16.66	0.097
Chronic obstructive lung disease (N, %)	10	20.83	8	21.62	2	11.11	0.616
Hyperlipidemia (N, %)	22	45.83	22	59.45	9	50	0.456
Cholesterol level (mmol/l, median, IQR)	4.2	3.45–5.15	4.25	3.5–5.27	4.4	3.75–5.65	0.864
Triglyceride level (mmol/l, median, IQR)	1.48	1.13–2.09	1.42	1.06–1.91	1.77	0.86–2.32	0.907
HDL cholesterol level (mmol/l, median, IQR)	1.21	1.07–1.72	1.27	1.12–1.52	1.24	1.15–1.30	0.944
LDL cholesterol level (mmol/l, median, IQR)	2.01	1.78–3.02	2.10	1.64–3.14	2.46	2.05–2.92	0.768
Statin use (N, %)	28	58.33	22	59.45	11	61.11	0.839
Thyroid dysfunction (N, %)	9	18.75	6	16.21	1	5.55	0.415
Peripheral arterial disease (N, %)	6	12.5	10	27.02	6	33.33	0.106
Operated side (LEFT) (N, %)	22	45.83	21	56.75	7	38.88	0.404
Degree of ICA stenosis (median, IQR)	80	80–90	80	80–90	80	80–90	0.985
Degree of the contralateral ICA stenosis(median, IQR)	40	40–67.5	40	10–60	42.5	0–70	0.968
Anatomy of CoW (complete, N, %)	18	37.5	8	21.62	6	33.33	0.224
Shunt use (N, %)	11	22.91	14	37.83	4	22.22	0.262
Time of the shunted period (sec, median, IQR)	34	25–40	30.5	22.5–38	37.5	29.25–47.25	0.405
Time of the clamping period (sec, median, IQR)	24	16–32.5	30	23–35	23	16.75–26.25	0.606
Median of the preclamp. rSO_2_ values (%, median, IQR)	72	62.25–77	68	61–75.5	69.75	62.62–80	0.19
Lowest rSO_2_ values during clamping period (%, median, IQR)	62.5	56–69	51	42–58.5	62.5	57.75–70.25	0.001
Median of the degree of desaturation (%, median, IQR)	12.41	7.47–16.13	25	17.24–29.33	11.39	8.25–14.44	0.001
Mean of MAP during clamping period (mean, SD)	88.12	10.65	87.73	9.83	85.61	8.09	0.655
Vasopressor use during the clamping period (N, %)	35	72.91	25	67.56	13	72.2	0.857
State Entropy median (median, IQR)	48.25	44–53.75	48	43–56.25	47	42–57.25	0.901
Response Entropy median (median, IQR)	50.25	47–56	50.50	46.75–58.5	52	45.37–59.12	0.904
Education (y, median, IQR)	12	11–15.75	12	11–15	11.5	11–14.5	0.742
MMSE test scores preoperatively (median, IQR)	29	27.25–30	29	27–29	28	26.75–29	0.143
MoCA test scores preoperatively (median, IQR)	28	26–29	28	27–29	25.5	22.75–26.25	0.001
Median time spent below 15.5% rSO_2_ reduction calculated from the baseline (median, IQR)	0	0–58.5	570	137–1214	0	0–0	0.001
Type of surgery (EEA, N, %)	37	77.08	23	62.16	14	77.77	0.219

*BMI* body mass index, *CoW* circle of Willis, *EEA* eversion endarterectomy, *HDL cholesterol* high-density lipoprotein cholesterol, *ICA* internal carotid artery, *IQR* interquartile range, *LDL cholesterol* low-density lipoprotein cholesterol, *MAP* mean arterial pressure, *MMSE* Mini Mental State Examination, *MoCA* Montreal Cognitive Assessment, *NCCF* group patients with no change in postoperative cognitive status proven by the MoCA, *PNCD group* patients with postoperative cognitive decline confirmed by the MoCA, *POCI group* patients with postoperative cognitive improvement confirmed by the MoCA rSO_2_: regional oxygen saturation, *SD* standard deviation, *Vascular POSSUM* Vascular-Physiological and Operative Severity Score for the enUmeration of Mortality and Morbidity

**Fig. 5:**
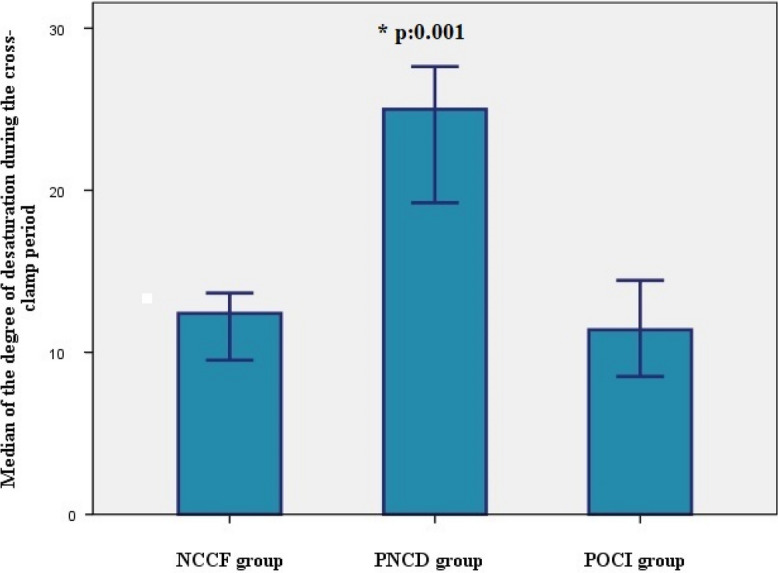
Comparison of the degree of desaturation between patients with different direction of cognitive change proven by the MoCA test. *NCCF group*: patients with no change in cognitive status proven by the MoCA, *PNCD group*: patients with postoperative cognitive decline confirmed by the MoCA, *POCI group*: patients with postoperative cognitive improvement confirmed by the MoCA

**Fig. 6:**
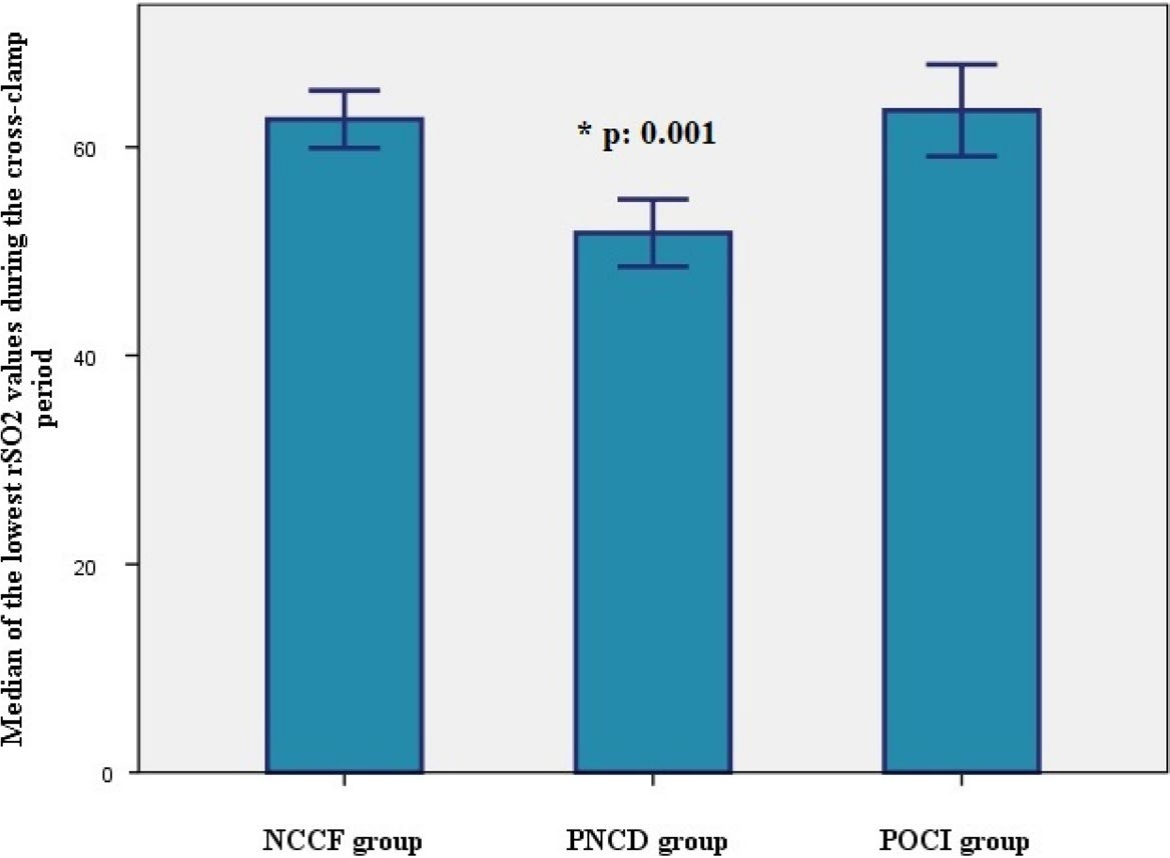
Comparison of the lowest rSO_2_ values between patients with different directions of cognitive change proven by the MoCA test. *NCCF group*: patients with no change in cognitive status proven by the MoCA, *PNCD group*: patients with postoperative cognitive decline confirmed by the MoCA, *POCI group*: patients with postoperative cognitive improvement confirmed by the MoCA

We found a higher proportion of women in the PNCD group. The degree of desaturation was significantly greater, and the lowest rSO_2_ values were also found in this group. The latter two differences are demonstrated in Figs. 5 and 6.

The results of the univariate analysis are shown in the Supplement (Tab s1.). In the multivariable model, female sex, diabetes, and degree of desaturation were independently associated with PNCD.

The preoperative MoCA scores were significantly lower in the POCI group, and the proportion of patients with diabetes was also smaller in this group. In the multivariable model, preoperative MoCA score and lower degree of desaturation were associated with POCI. The results are shown in Table 3.

**Table 3:** The results of the multivariable logistic regression analysis

Characteristic	PNCD group				POCI group			
	OR	95% C.I		*p* value	OR	95% C.I		*p* value
		Lower	Upper			Lower	Upper	
Sex	3.438	1.096	10.779	0.034	−			
Vascular Possum score	1.050	0.905	1.218	0.522	−			
Non-insulin-dependent diabetes mellitus	3.809	1.168	12.422	0.027	−			
Median of the degree of desaturation	1.184	1.070	1.310	0.001	0.896	0.804	0.998	0.046
Preoperative MoCA score	−				0.588	0.426	0.814	0.001

*C.I.* confidence interval, *MoCA* Montreal Cognitive Assessment, *OR* odds ratio, *PNCD group* patients with postoperative cognitive decline confirmed by the MoCA, *POCI group* patients with postoperative cognitive improvement confirmed by the MoCA, *Vascular POSSUM* Vascular-Physiological and Operative Severity Score for the enUmeration of Mortality and Morbidity

### Impact of age on cerebral tissue saturation and on the change of cognitive function

Analyzing the impact of age on our results, we divided our patients into four groups, based on their age: group I—7 patients (5.9%), younger than 60 years, group II—43 patients (36.4%), aged 60–69 years, group III—42 patients (35.6%), aged 70–79, group IV—11 patients (9.3%), older than or equal to 80 years.

There was no significant difference between the groups regarding cerebral tissue saturation values and the change in the scores of the cognitive tests. However, older patients gained lower scores on the preoperative and postoperative cognitive tests, whose result could not be linked to the different levels of education.

Smoking was more frequent among younger patients (*p*: 0.008), while the incidence of COPD (chronic obstructive pulmonary disease) was higher in the oldest group (*p*: 0.039). BMI (body mass index) proved to be higher in the “younger” population (*p*: 0.030).

Though it did not reach significant level (*p*: 0.062), the incidence of hyperlipidemia was the highest among the youngest patients of our study. Proceeding from this result, we analyzed the levels of lipid parameters and have found a statistically significant difference regarding the levels of cholesterol. The difference regarding triglyceride levels did not reach the level of significance (*p*: 0.056), but they were considerably higher among the youngest patients of the study. The results are shown in Table 4.

**Table 4:** Comparison of clinical factors between the four groups created based on the classification of ages

Characteristic	Group I (≤ 59 years) 7 patients (5.9%)	Group II (60–69 years) 43 patients (36.4%)	Group III (70–79 years) 42 patients (35.6%)	Group IV (≥ 80 years) 11 patients (9.3%)	*p* value
MMSE test scores preoperatively (median, IQR)	29 (28–30)	29 (28–30)	28 (27–29)	27 (27–29)	0.040
MoCA test scores preoperatively (median, IQR)	29 (26–30)	28 (26–29)	28 (25–28)	25 (24–26)	0.001
MMSE test scores postoperatively (median, IQR)	29 (28–30)	29 (28–30)	29 (28–30)	27 (25–29)	0.003
MoCA test scores postoperatively (median, IQR)	28 (27–29)	27 (25–28)	26.5 (24–28)	24 (24–26)	0.002
Change in the MMSE scores (median, IQR)	0 (− 1 to 0)	0 (0–1)	0 (0–2)	0 (–2 – 1)	0.312
Change in the MoCA scores (median, IQR)	0 (− 2 to 1)	− 1 (− 2 to 1)	− 0.5 (− 2 to 1.25)	0 (− 2 to 1)	0.975
Education (y, median, IQR)	11 (11–12)	12 (11–16)	12 (11–16)	12 (10–16)	0.951
Median of the preclamp. rSO_2_ values (%, median, IQR)	74 (64–86)	70 (60–77)	71 (65.5–77)	65 (64–75)	0.590
Lowest rSO_2_ values during clamping period (%, median, IQR)	67 (54–72)	60 (50–67)	57 (51–64.62)	57 (51–66)	0.552
Median of the degree of desaturation (%, median, IQR)	13.51 (11.39–16.27)	14.28 (9.85–22.03)	15.04 (9.41–26.59)	13.66 (11.33–18.42)	0.836
Chronic Obstructive lung disease (N, %)	0 (0)	10 (23.25)	5 (11.9%)	5 (45.45)	0.039
BMI (median, IQR)	28.4 (23.33–31.6)	29.06 (27.04–30.86)	27.22 (23.95–29.21)	26.72 (22.22–29.01)	0.030
Smoker (N, %)	3 (42.85)	20 (46.51)	6 (14.28)	2 (18.18)	0.008
Hyperlipidemia (N, %)	5 (71.42)	26 (60.46)	15 (35.71)	7 (63.63)	0.062
Statin use (N, %)	3 (42.85)	22 (51.16)	28 (66.66)	10 (90.90)	0.059
Cholesterol (mmol/l, median, IQR)	5.1 (4.77–6.75)	3.95 (3.28–5.22)	4.4 (3.7–5.5)	4.1 (3.6–4.3)	0.042
Triglycerides (mmol/l, median, IQR)	2.85 (1.25–3.15)	1.5 (1.21–2.42)	1.4 (1– 1.90)	1.25 (0.83–1.7)	0.056
HDL cholesterol (mmol/l, median, IQR)	1.3 (1.05–1.78)	1.20 (1.01–1.39)	1.31 (1.2–1.73)	1.14 (0.94–1.5)	0.207
LDL cholesterol (mmol/l, median, IQR)	3.3 (2.52–4.35)	1.95 (1.59–3.02)	2.28 (1.92–3.12)	1.96 (1.69–2.61)	0.092

*BMI* body mass index, *HDL cholesterol* high-density lipoprotein cholesterol, *IQR* interquartile range, *LDL cholesterol* low-density lipoprotein cholesterol, *MMSE* Mini Mental State Examination, *MoCA* Montreal Cognitive Assessment, *rSO*_*2*_ regional oxygen saturation, *SD* standard deviation

## Discussion

In our prospective study, we aimed to analyze the relationship between cerebral tissue saturation during carotid endarterectomy and postoperative cognitive function.

We found a significant correlation between the change in the MoCA scores compared to preoperative values and cerebral tissue oxygen desaturation. There was no significant relationship with changes in the MMSE score. Similarly, the correlation between the lowest rSO_2_ values and the difference in the preoperative and postoperative MoCA scores was significant but did not show any significant result in the case of the MMSE score.

We found that the percent change of the cerebral saturation values compared to the preclamping values was a better predictor than the absolute lowest saturation values. A desaturation of 15.5% was associated with postoperative cognitive decline, and a desaturation of less than 12.5% was associated with cognitive improvement.

MoCA and MMSE are widely used screening tools for the detection of cognitive impairment. Both tests have been proven to be accurate in detecting AD [19]. The most crucial difference between the two tests is the ability to detect subtle changes in cognitive function. MoCA has been proven to be able to detect early signs of cognitive impairment, while MMSE has a low sensitivity in this regard. [19–22]. Besides, MMSE has been criticized for being less capable of assessing the safety of executive functions [23].

In our study, MMSE failed to show significant correlation neither with the ratio of desaturation nor with the lowest saturation value, while MoCA showed a good correlation with the cognitive changes. Therefore, MoCA seems to measure the changes in cognitive performance more accurately in the perioperative period. In accordance with previous studies and based on our results, we recommend the use of the MoCA test in perioperative circumstances.

We used NIRS and Entropy monitoring to track cerebral function. The baseline differences in the NIRS values can be due to biological variations in the cerebral arterial/venous ratio and the effect of age and sex on cerebral rSO_2_. Therefore, the value of near-infrared spectroscopy lies in the monitoring of the trend of the changes in cerebral rSO_2_ [24, 25]. Our results also supported the rationale of trend monitoring, i.e., the degree of cerebral tissue desaturation has a higher predictive value than the absolute value of saturation.

The lowest rSO_2_ value and the degree of desaturation were associated with postoperative cognitive decline. This relationship has been previously discussed in other fields of medicine, such as cardiac and thoracic or orthopedic surgery [26–29].

The threshold of critical cerebral tissue saturation varies between a drop of 10 and 20% [8, 9, 30]. A 20% decrease in rSO_2_ was found to cause an eightfold increase in the development of cognitive disorders [31]. In one study, the allowed 20% drop from baseline did not reduce the occurrence of PNCD. They recommended a higher absolute value to prevent the occurrence of PNCD [32]. Our result at 15.5% desaturation is similar to other studies. The predictivity of the absolute value of desaturation (57.7%) was similar to the data reported in other studies [32, 33].

The link between improvement in cognitive performance and cerebral tissue saturation during carotid surgery has not been examined extensively. The importance of stable perioperative hemodynamic parameters in the late improvement in cognitive performance has been discussed in a few studies [34]. Prevention of a significant drop in cerebral rSO_2_ values might have a positive effect on postoperative cognitive performance, but at least it can be used as a predictive factor for developing POCI [35]. Based on our study, defining an absolute value of minimum rSO_2_ at 60.75% as a limit for postoperative improvement seems to be a reliable threshold and is in acceptable accordance with previous studies [33].

Besides that female sex was an independent risk factor for postoperative cognitive decline, significant desaturation occurred more frequently in women, but the anatomy of the circle of Willis and the degree of stenosis were similar.

The prevalence of mild cognitive impairment is higher among women, and the progression of the decline is also faster [36, 37]. Sex differences might be explained by different brain and cognitive reserves, which might have an effect on tolerance to surgical strain and hemodynamic changes [38] [39].

Cognitive improvement was associated with lower preoperative MoCA scores among those who showed improvement in cognitive performance postoperatively. In a similar study, younger age and lower preoperative MoCA scores were strong predictors of cognitive improvement after carotid endarterectomy [40].

The finding of a higher proportion of patients with diabetes in the PNCD group might be explained by atherosclerotic changes in small- and medium-sized arteries and that these vessels are more vulnerable to surgical stress and hemodynamic changes [41]. There are controversial data in the literature regarding the risk associated with diabetes after carotid endarterectomies [42, 43].

As age is considered an important risk factor for neurologic complications after surgical interventions, we aimed to analyze the impact of age on cerebral tissue saturation and cognitive function. Intriguingly, we have not found any statistically significant difference in this relation, apart from lower cognitive performance observed in older patients.

As normal aging has an adverse impact on cognitive abilities, lower performance among older patients is not a novelty. Age-associated cognitive decline may be explained among others with changes in white matter, a decrease in white and grey matter volume, and changes in neurotransmitter levels, which are part of the normal cognitive aging. Some mental functions are less affected by this process, whereas in the case of other cognitive abilities, such as memory, processing speed, reasoning, and executive functions, this decline is more perceptible. Applying cognitive training may have a favorable effect to preserve cognitive abilities with advancing age [44].

However, there was no significant difference between the groups created based on the classification of ages neither regarding cerebral tissue saturation values and the degree of desaturation nor in the change of cognitive tests.

Significantly higher BMI and cholesterol levels and higher frequency of smoking found among our youngest patients may explain this result.

The condition of the cardiovascular system, the extent of functional deterioration is highly variable among individuals of the same chronological age.

Elevated lipid levels, obesity, and smoking may contribute to the early impairment of the cardiovascular system, making it more vulnerable to hemodynamic changes and surgical stress than it would be expected based on the chronological age of the patient. [45–49]. Cardiovascular risk is higher in case of these patients, despite their relatively young chronological age.

### Limitations

Our study has several limitations, such as the lack of a control group and the limited ability of NIRS to detect changes that are not located in the forebrain. Although it can be said that postoperative cognitive assessment occurred in a stable phase, for the sake of completeness, we have to mention that these changes might alter over time. Repeated assessment is necessary so that these subtle changes can be considered definitive [50]. Our study was a single-center prospective study of patients treated under general anesthesia. The COVID epidemic has also contributed to a less effective follow-up.

## Conclusion

In our study, we found a correlation between cerebral tissue desaturation measured by NIRS and postoperative changes in cognitive function measured by the MOCA.

We found that desaturation higher than 15.5% during cross-clamping was associated with cognitive decline. In contrast, a desaturation less than 12.5% was associated with cognitive improvement. Moreover, women and patients with diabetes must be monitored and followed more closely because they seem to be more vulnerable during carotid surgery.

Although these results of postoperative cognitive performance might not be explained completely by the changes in cerebral tissue saturation, our results suggest its determining role in these trends. Therefore, it must be taken into account when performing carotid surgery to benefit the patients’ overall well-being.

## Supplementary Information

Below is the link to the electronic supplementary material.

Supplementary file1 (DOCX 111 KB)

## Data Availability

The data that support the findings of this study are available from the corresponding author, Andrea Székely, upon reasonable request.

## Abbreviations

*ASA score*: American Society of Anesthesiologists physical status classification
*BMI*: Body mass index
*CCA*: Common carotid artery
*C.I.*: Confidence interval
*COPD*: Chronic obstructive pulmonary disease
*CoW*: Circle of Willis
*EEA*: Eversion endarterectomy
*HDL cholesterol*: High-density lipoprotein cholesterol
*ICA*: Internal carotid artery
*IQR*: Interquartile range
*LDL cholesterol*: Low-density lipoprotein cholesterol
*MAP*: Mean arterial pressure
*MMSE*: Mini Mental State Examination
*MoCA*: Montreal Cognitive Assessment
*NASCET*: The North American Symptomatic Carotid Endarterectomy Trial
*NCCF group*: Patients with no change in postoperative cognitive status proven by the MoCA
*NIRS*: Near-infrared spectroscopy
*OR*: Odds ratio
*PNCD*: Perioperative neurocognitive disorder
*PNCD group*: Patients with postoperative cognitive decline confirmed by the MoCA
*POCI*: Postoperative cognitive improvement
*POCI group*: Patients with postoperative cognitive improvement confirmed by the MoCA
*PWV*: Pulse wave velocity
*rSO*_*2*_: Regional cerebral oxygen saturation
*rSO*_*2desat*_: Median of the degree of regional cerebral oxygen saturation desaturation
*rSO*_*2low*_: Lowest rSO_2_ values during clamping period
*SD*: Standard deviation
*Vascular POSSUM*: Vascular-Physiological and Operative Severity Score for the enUmeration of Mortality and Morbidity
