# Supplementary material for: Cerebrovascular dysregulation and postoperative cognitive alterations after carotid endarterectomy
Source: GeroScience. 2024 Jun 15;46(6):6301–15. doi: 10.1007/s11357-024-01237-6 (PMC11493908; doi:10.1007/s11357-024-01237-6)
Supplement: Supplementary file 1 — Supplementary file1 (DOCX 111 KB) [file 11357_2024_1237_MOESM1_ESM.docx]

Cerebrovascular dysregulation and postoperative cognitive alterations after carotid endarterectomy

Á. D. Sándor^1^, Zs. Czinege^2^, A. Szabó^3^, E. Losoncz^4^, K. Tóth^4^, Zs. Mihály^2^, P. Sótonyi^2^, B. Merkely^5^, A. Székely^1*^

**Supplement**


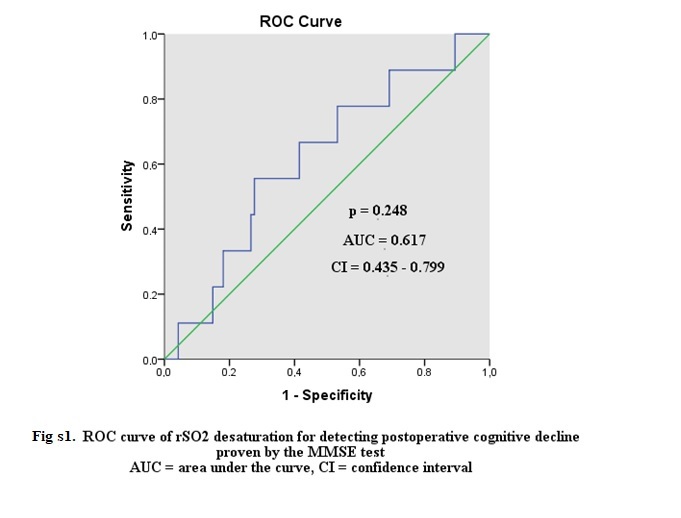


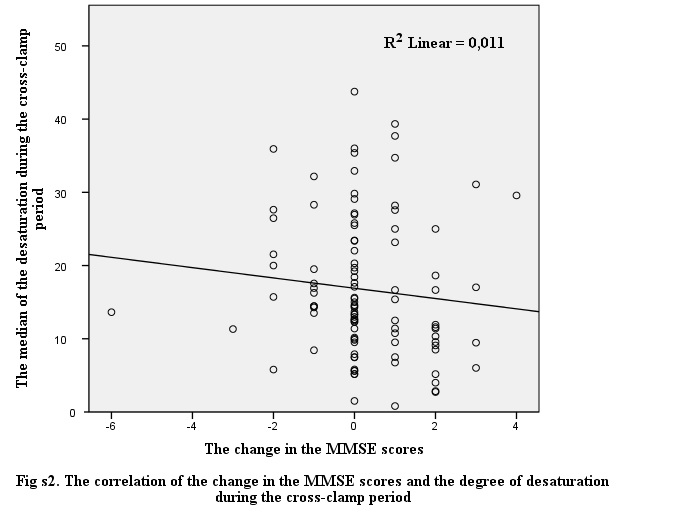


| Characteristic | PNCD group | | | | POCI group | | | |
| --- | --- | --- | --- | --- | --- | --- | --- | --- |
|  | OR | 95% C.I | | Sig. | OR | 95% C.I.) | | Sig. |
|  |  | Lower | Upper |  |  | Lower | Upper |  |
| Age | 1.025 | 0.969 | 1.086 | 0.389 | 1.037 | 0.964 | 1.115 | 0.324 |
| Gender | 3.520 | 1.516 | 8.174 | 0.003 | 0.618 | 0.212 | 1.081 | 0.378 |
| Mean of MAP during the clamping period | 1.003 | 0.963 | 1.045 | 0.884 | 0.976 | 0.926 | 1.029 | 0.363 |
| Use of vasopressor | 0.781 | 0.325 | 1.876 | 0.581 | 1.083 | 0.349 | 3.360 | 0.890 |
| Median of the lowest rSO2 | 0.881 | 0.833 | 0.932 | 0.000 | 1.052 | 1.000 | 1.106 | 0.050 |
| Median of the desaturation | 1.199 | 1.117 | 1.288 | 0.000 | 0.883 | 0.811 | 0.962 | 0.004 |
| ASA score | 1.107 | 0.496 | 2.470 | 0.804 | 1.189 | 0.429 | 3.301 | 0.739 |
| VascularPossum score | 1.099 | 0.986 | 1.225 | 0.088 | 1.023 | 0.896 | 1.168 | 0.736 |
| Hypertension | 0.929 | 0.209 | 4.128 | 0.923 | 1.526 | 0.176 | 13.229 | 0.701 |
| Ischaemic heart disease | 0.960 | 0.407 | 2.263 | 0.926 | 0.743 | 0.241 | 2.287 | 0.604 |
| COPD | 1.241 | 0.456 | 3.381 | 0.672 | 0.465 | 0.098 | 2.213 | 0.336 |
| Diabetes mellitus type 2 | 2.261 | 0.930 | 5.498 | 0.072 | 0.126 | 0.016 | 0.999 | 0.050 |
| Diabetes mellitus type- 1 | 2.361 | 0.668 | 8.350 | 0.183 | 1.056 | 0.208 | 5.357 | 0.948 |
| Diabetes mellitus (type 1 + type 2) | 2.103 | 0.910 | 4.860 | 0.082 | 0.315 | 0.085 | 1.173 | 0.085 |
| Neurological disorder | 0.793 | 0.291 | 2.165 | 0.651 | 0.406 | 0.086 | 1.920 | 0.256 |
| Previous stroke | 0.938 | 0.355 | 2.478 | 0.897 | 0.992 | 0.292 | 3.371 | 0.990 |
| Hyperlipidaemia | 1.656 | 0.733 | 3.741 | 0.225 | 0.932 | 0.337 | 2.577 | 0.892 |
| Thyroid gland disorder | 1.084 | 0.360 | 3.266 | 0.886 | 0.275 | 0.034 | 2.225 | 0.226 |
| Peripheral arterial disease | 1.667 | 0.639 | 4.344 | 0.296 | 2.156 | 0.703 | 6.614 | 0.179 |
| Smoking (current) | 0.973 | 0.404 | 2.344 | 0.951 | 0.386 | 0.103 | 1.443 | 0.157 |
| Alcohol consumption | 0.320 | 0.066 | 1.547 | 0.156 | 1.689 | 0.409 | 6.981 | 0.469 |
| BMI | 1.046 | 0.957 | 1.143 | 0.318 | 1.001 | 0.895 | 1.119 | 0.989 |
| Education (yrs) | 1.087 | 0.924 | 1.278 | 0.313 | 0.934 | 0.756 | 1.154 | 0.527 |
| Time of the cross clamp | 1.033 | 0.975 | 1.094 | 0.267 | 0.982 | 0.939 | 1.027 | 0.420 |
| shunt | 2.070 | 0.859 | 4.985 | 0.105 | 0.686 | 0.205 | 2.288 | 0.539 |
| Operated side | 0.597 | 0.265 | 1.345 | 0.213 | 1.609 | 0.569 | 4.546 | 0.370 |
| Degree of the stenosis on the operated side | 1.016 | 0.962 | 1.073 | 0.568 | 0.979 | 0.917 | 1.045 | 0.526 |
| Degree of the stenosis on the contralat. side | 1.001 | 0.988 | 1.013 | 0.889 | 0.998 | 0.983 | 1.014 | 0.834 |
| Anatomy of Circle of Willisii | 2.344 | 0.750 | 7.324 | 0.143 | 0.653 | 0.173 | 2.460 | 0.529 |
| **Tab s1. Results of the univariate logistic regression analysis for all variable**  OR: odds ratio, C.I.: confidence interval, ASA score: American Society of Anaesthesiologists physical status classification**,** BMI: body mass index, COPD: Chronic obstructive pulmonary disease, MAP: mean arterial pressure, Vascular POSSUM: Vascular-Physiological and Operative Severity Score for the enUmeration of Mortality and Morbidity | | | | | | | | |
